# Small extracellular vesicle-mediated *ITGB6* siRNA delivery downregulates the αVβ6 integrin and inhibits adhesion and migration of recipient prostate cancer cells

**DOI:** 10.1080/15384047.2022.2030622

**Published:** 2022-02-19

**Authors:** Shiv Ram Krishn, Vaughn Garcia, Nicole M. Naranjo, Fabio Quaglia, Christopher D. Shields, Maisha A. Harris, Andrew V. Kossenkov, Qin Liu, Eva Corey, Dario C. Altieri, Lucia R. Languino

**Affiliations:** a Prostate Cancer Discovery and Development Program, Thomas Jefferson University, Philadelphia, PA USA; b Department of Cancer Biology, Sidney Kimmel Cancer Center, Thomas Jefferson University, Philadelphia, PA USA; c Center for Systems and Computational Biology, the Wistar Institute, Philadelphia, PA USA; d Molecular and Cellular Oncogenesis Program, the Wistar Institute, Philadelphia, PA USA; e Department of Urology, University of Washington, Seattle, WA USA; f Prostate Cancer Discovery and Development Program, the Wistar Institute, Philadelphia, PA USA; g Immunology, Microenvironment and Metastasis Program, the Wistar Institute, Philadelphia, PA USA

**Keywords:** Adhesion, androgen receptor, electroporation, integrin, migration, prostate cancer, siRNA, small extracellular vesicles, therapy

## Abstract

The αVβ6 integrin, an epithelial-specific cell surface receptor absent in normal prostate and expressed during prostate cancer (PrCa) progression, is a therapeutic target in many cancers. Here, we report that transcript levels of *ITGB6* (encoding the β6 integrin subunit) are significantly increased in metastatic castrate-resistant androgen receptor-negative prostate tumors compared to androgen receptor-positive prostate tumors. In addition, the αVβ6 integrin protein levels are significantly elevated in androgen receptor-negative PrCa patient derived xenografts (PDXs) compared to androgen receptor-positive PDXs. *In vitro*, the androgen receptor-negative PrCa cells express high levels of the αVβ6 integrin compared to androgen receptor-positive PrCa cells. Additionally, expression of androgen receptor (wild type or variant 7) in androgen receptor-negative PrCa cells downregulates the expression of the β6 but not αV subunit compared to control cells. We demonstrate an efficient strategy to therapeutically target the αVβ6 integrin during PrCa progression by using short interfering RNA (siRNA) loaded into PrCa cell-derived small extracellular vesicles (sEVs). We first demonstrate that fluorescently-labeled siRNAs can be efficiently loaded into PrCa cell-derived sEVs by electroporation. By confocal microscopy, we show efficient internalization of these siRNA-loaded sEVs into PrCa cells. We show that sEV-mediated delivery of *ITGB6*-targeting siRNAs into PC3 cells specifically downregulates expression of the β6 subunit. Furthermore, treatment with sEVs encapsulating *ITGB6* siRNA significantly reduces cell adhesion and migration of PrCa cells on an αVβ6-specific substrate, LAP-TGFβ1. Our results demonstrate an approach for specific targeting of the αVβ6 integrin in PrCa cells using sEVs encapsulating *ITGB6*-specific siRNAs.

## Introduction

Prostate cancer (PrCa) is currently the second leading cause of estimated cancer-related death in men in the US.^1^ Patients with metastatic PrCa disease exhibit much less favorable outcomes with a 5-year survival rate of 30%.^2^ The development of resistance to current therapeutic modalities in most advanced lethal PrCa patients underscores the need for novel therapeutic targets and strategies.^3,4^ Furthermore, developing personalized treatment methods for PrCa patients necessitates understanding the expression pattern of differentially regulated genes and proteins in prostate tumor and their impact on prostate tumor biology. Therapeutic targeting in PrCa could then be directed against molecules that are overexpressed in transformed cells relative to healthy tissue. One such potential target is the epithelial-specific αVβ6 integrin.^5–7^The physiologic expression of the αVβ6 integrin is restricted to development and epithelial re-modeling during tissue repair.^5^ In contrast, its expression is upregulated in organ fibrosis^8–12^ and solid tumors derived from breast, lung, liver, stomach, pancreas, colon, cervix, and ovary, where it is generally associated with poorer prognosis.^13–20^ The αVβ6 integrin activates latent transforming growth factor beta 1 (TGFβ1), promotes epithelial-to-mesenchymal transition, cellular migration, and matrix metalloproteinase activity in cancer.^13,15,21,22^ In light of these attributes, the αVβ6 integrin has been targeted for the treatment of different cancers using diverse strategies.^18,19^ Our group has demonstrated that expression of the αVβ6 integrin is not detectable in normal human prostate; however, it is highly expressed in human primary PrCa,^23^ PrCa bone metastases,^24^ and peripheral blood mononuclear cells (PBMC) from PrCa patients.^7^ The αVβ6 integrin expression plays an important role in prostate tumor progression by promoting colony formation,^25^ cell adhesion, cell migration on an αVβ6-specific substrate, the latency-associated peptide (LAP)-TGFβ,^26^ as well as activation of an osteolytic program by inducing matrix degradation through MMP2.^24^ Furthermore, PrCa cell-derived small extracellular vesicles (sEVs) play an important role in prostate tumor microenvironment;^27,28^ transfer of αVβ6 integrin via PrCa cell-derived sEVs results in increased migration of recipient PrCa cells,^6^ M2 polarization of monocytes,^7^ and angiogenesis.^29^

To investigate the potential therapeutic utility ofαVβ6 integrin targeting in PrCa, our group has previously established that treatment of Pten^pc-/ –^ mice containing a prostate-specific deletion of the Pten tumor suppressor with 6.3G9, an anti-αVβ6 non-ligand-mimetic blocking monoclonal antibody that is not internalized upon binding,^30^ results in a significant decrease in prostate tumor weight.^23^ Despite the rapid progress made toward the development of potent anti-cancer therapeutic antibodies, challenges remain to overcome their limitations, including side effects, immunogenicity, low efficacy due to resistance to therapy, access to targets, the complexity of biological systems, and individual variations.^31^ Therefore, tissue-specific, nontoxic, and non-immunogenic delivery technologies are critical to move therapeutic modalities into clinical practice for cancer therapy. In this context, recent reports have shown the therapeutic utility of exosomes.^32–35^ Exosomes are sEVs of endosomal origin that are released from all cells.^27,33,36^ The current sEV isolation protocols typically purify a mixture of endosomal (namely, exosomes) and non-endosomal sEVs.^37^ Therefore, for the purposes of this study, sEVs are defined as a population of EVs recovered by 100,000 x*g* ultracentrifugation followed by iodixanol density gradient isolation, <200 nm in size, endosomal or non-endosomal in origin, and secreted by fusion with the plasma membrane.^36–38^ sEVs carry RNA, DNA, and proteins from their cells of origin, are captured and internalized by recipient cells, and can modulate recipient cell phenotypes by delivering their cargo.^27,28^ sEVs can be engineered to deliver short interfering RNAs (siRNAs), antisense oligonucleotides, antibodies, or chemotherapeutic drugs to a desired target.^33,39^ It has been reported that delivery of sEVs loaded with siRNAs to target cancer tissues/cells can accomplish specific gene knockdown and inhibit tumor growth in mouse models.^34,35,40^

Based on these previous findings, we hypothesize that inhibition of the β6 subunit expression in PrCa cells through sEV-mediated delivery of siRNAs against *ITGB6* might consequently impact the functions of the αVβ6 integrin in PrCa cells. Here, we demonstrate an efficient strategy to therapeutically target the αVβ6 integrin during PrCa progression by using siRNAs loaded into PrCa cell-derived sEVs. Upon internalization, these sEVs deliver *ITGB6*-targeting siRNAs to recipient PrCa cells, thereby inhibiting expression of the β6 subunit and significantly reducing cell adhesion and migration of the cells on the αVβ6 integrin specific substrate, LAP-TGFβ1. Taken together, our results support the feasibility of using sEVs bearing *ITGB6* siRNA to directly modulate expression of the αVβ6 integrin as a potential therapeutic strategy for PrCa.

## Results

### αVβ6 integrin expression negatively correlates with androgen receptor levels in prostate cancer.

**Figure 1.**
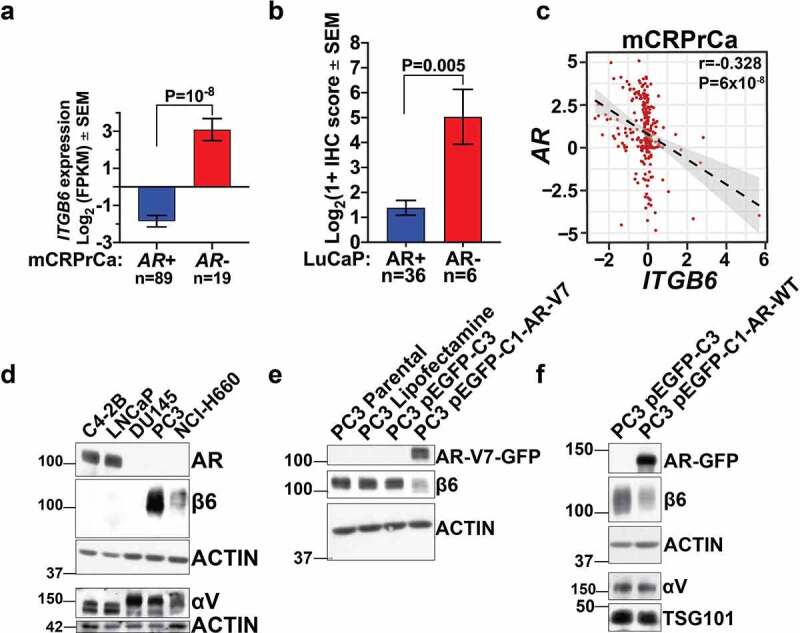
**αVβ6 integrin expression and its correlation with androgen receptor in prostate cancer**. (a) RNAsequencing analysis of metastatic castrate-resistant prostate cancer (mCRPrCa) specimens acquired through rapid autopsy.^41^ Specimens are classified based on their levels of androgen receptor (*AR*). The bar graphs show *ITGB6* expression as Log base 2 fragments per kilobase of transcript per million mapped reads (FPKM) in *AR*-positive (*AR+*, n = 89) and *AR*-negative (*AR-*, n = 19) cases. The values are presented as mean ± SEM; P values were calculated by the Mann-Whitney non-parametric test. (b) Analysis of the immunohistochemical (IHC) results of αVβ6 integrin expression in 42 LuCaP PDX models^42,43^ classified based on expression of AR. Bar graphs show the Log base 2 (1+ IHC score) for αVβ6 integrin expression in AR*+* (N = 36) and AR – (N = 6) LuCaP PDXs. The values are presented as mean ± SEM; P values were calculated by using the Mann-Whitney non-parametric test. (c) Correlation between *ITGB6* and *AR* expression in mCRPrCa cases acquired using the Prostate Cancer Transcriptome Atlas web tool.^44^ The scatter plot with linear regression line represents the normalized *AR* expression values on the ordinate and, on the abscissa, the normalized *ITGB6* expression values in mCRPrCa cases. P-values were calculated using the correlation test R function. (d) IB analysis for expression of AR, β6 subunit, ACTIN as loading control (reducing conditions), and αV, ACTIN as loading control (non-reducing conditions) in total cell lysates (TCL) from C4-2B, LNCaP, DU145, PC3, and NCI-H660 PrCa cells. (e) IB analysis (reducing conditions) for expression of AR-V7, β6 subunit and ACTIN as loading control in total cell lysates from PC3 parental cells, PC3 cells treated with lipofectamine, transiently transfected with pEGFP-C3 empty vector, or the pEGFP-C1-AR-V7 vector. (f) IB analysis for expression of AR, β6 subunit and ACTIN as loading control (reducing conditions) and αV subunit, TSG101 as loading control (non-reducing conditions) in total cell lysates from PC3 cells transiently transfected with pEGFP-C3 empty vector or pEGFP-C1-AR-WT vector.

Our group has previously reported that expression of the αVβ6 integrin in the LNCaP PrCa cell line causes an increase in androgen receptor (AR) activity without inducing changes in AR proteins’ endogenous expression.^23^ However, inversely, the impact of AR expression on the αVβ6 integrin expression has never been explored. To address this issue, we first interrogated the RNA-sequencing dataset^41^ on metastatic castrate-resistant prostate cancer (mCRPrCa) specimens and classified them according to their presence or absence of *AR* expression. Our analysis shows a significantly increased expression of the *ITGB6* transcript, which encodes the β6 integrin subunit, in *AR*-negative tumors (n = 19, P = 10,^−8^ Mann-Whitney test) compared to *AR*-positive tumors (n = 89) (Figure 1a). To corroborate these findings from RNA-sequencing data, we classified the LuCaP patient-derived xenografts (PDXs)^41,42^ based on presence or absence of AR expression, and compared their αVβ6 integrin expression levels as determined by immunohistochemistry (IHC), previously published by our group.^43^ Our data demonstrate a significantly increased expression of the αVβ6 integrin in AR-negative LuCaP PDXs (n = 6, P = .005, Mann-Whitney test) compared to AR-positive PDXs (n = 36) (Figure 1b). Moreover, the analysis of the mRNA dataset from the Prostate Cancer Transcriptome Atlas (PCTA) web tool^44^ shows a significant negative correlation between mRNA expression of *ITGB6* and *AR* in mCRPrCa cases (r = −0.328, P = 6x10,^−8^ Spearman correlation). Additionally, immunoblotting (IB) analysis reveals that the β6 subunit is not expressed in AR-positive cell lines (C4-2B, LNCaP), whereas it is highly expressed in AR-negative PrCa cell lines (PC3 and NCI-H660) (Figure 1d). Furthermore, the DU145 PrCa cell line does not express either AR or the β6 subunit. Our IB data also demonstrate that the αV subunit is expressed in all PrCa cells analyzed, irrespective of their AR expression status (Figure 1d). The AR splice variant 7 (AR-V7), has emerged as a biomarker for mCRPrCa.^45^ Therefore, we next investigated the β6 subunit levels upon transient expression of AR-V7 in PC3 cells compared to control AR-V7 negative PC3 cells. By IB analysis, our data demonstrate a reduction in the expression of β6 subunit in PC3 cells that transiently express AR-V7 (Figure 1e) or full-length AR (AR-WT; Figure 1f)but not αV subunit compared to control AR-negative PC3 cells. Overall, our data are consistent with a central role for AR loss on αVβ6 integrin expression during PrCa progression.

### Small extracellular vesicles loaded with fluorescently-labeled siRNAs are efficiently internalized into prostate cancer cells.

**Figure 2.**
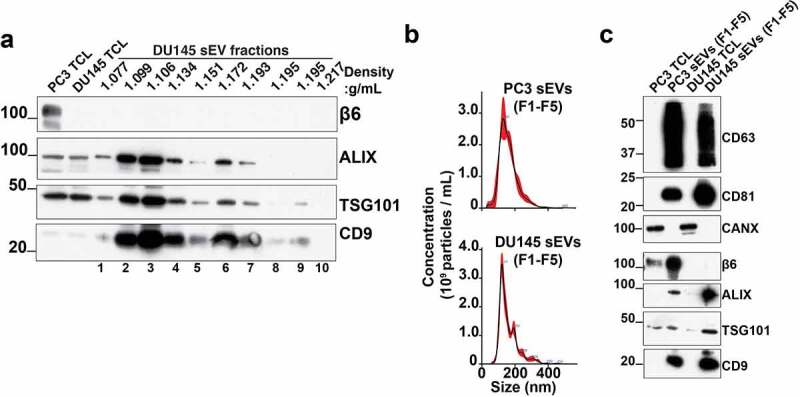
**Characterization of prostate cancer cell-derived small Extracellular Vesicles (sEVs)**. (a) IB analysis (reducing conditions) for expression of β6 subunit, ALIX, TSG101, CD9, in PC3 total cell lysate, DU145 total cell lysate (TCL) and lysates from density gradient-isolated DU145 small extracellular vesicles (sEVs) in fractions one to ten. (b) Nanoparticle tracking analysis (NTA) of the PC3 and DU145 cell-derived, density gradient-isolated sEVs pooled from fractions one to five (sEV F1-F5). (c) IB analysis for expression of CD63, CD81, CANX (non-reducing conditions), and β6 subunit, ALIX, TSG101, CD9 (reducing conditions) in PC3 TCL, PC3 sEVs (F1-F5), DU145 TCL and DU145 sEVs (F1-F5).

Since αVβ6 integrin is highly expressed during progression of PrCa,^23,24^ we explored the therapeutic utility of *ITGB6* siRNA-loaded small extracellular vesicles (sEVs) on PrCa cells. For exogenous loading of siRNAs, sEVs were isolated from serum-starved conditioned media from PrCa cells (DU145 and PC3) by high-speed differential ultracentrifugation (100,000 x*g*). To further remove protein and non-vesicular contaminants, we performed density gradient isolation of sEVs.^29^ The DU145 cell-derived sEVs were floated on an iodixanol density gradient and sEVs in fractions one to ten were characterized by IB. Our IB analysis shows that β6 subunit expression is observed only in PC3 TCL whereas it is absent in DU145 TCL; the sEV markers ALIX, TSG101, CD9 are expressed in iodixanol density gradient isolated fractions, whereas β6 subunit expression is absent in all iodixanol density gradient isolated, DU145-derived, sEV fractions (Figure 2a). We then pooled the sEVs floating in fractions one to five (F1-F5) corresponding to density 1.077–1.151 g/mL and characterized by nanoparticle tracking analysis (NTA). The NTA data from PC3 and DU145 sEVs (F1-F5) show that the majority of the sEVs exhibit a particle size of <150 nm (Figure 2b). The pooled iodixanol density gradient purified sEVs were further characterized by IB for expression of the β6 subunit and sEV markers. Our IB analysis on TCL and sEVs (F1-F5) from PC3 and DU145 cells shows expression of the β6 subunit only in PC3 TCL and sEVs. The sEV markers CD63, CD81, ALIX, TSG101 and CD9 are expressed in sEVs (F1-F5) (Figure 2c) whereas the endoplasmic reticulum marker calnexin (CANX) is only expressed in TCL but not in sEVs derived from both PC3 and DU145 cells.

**Figure 3.**
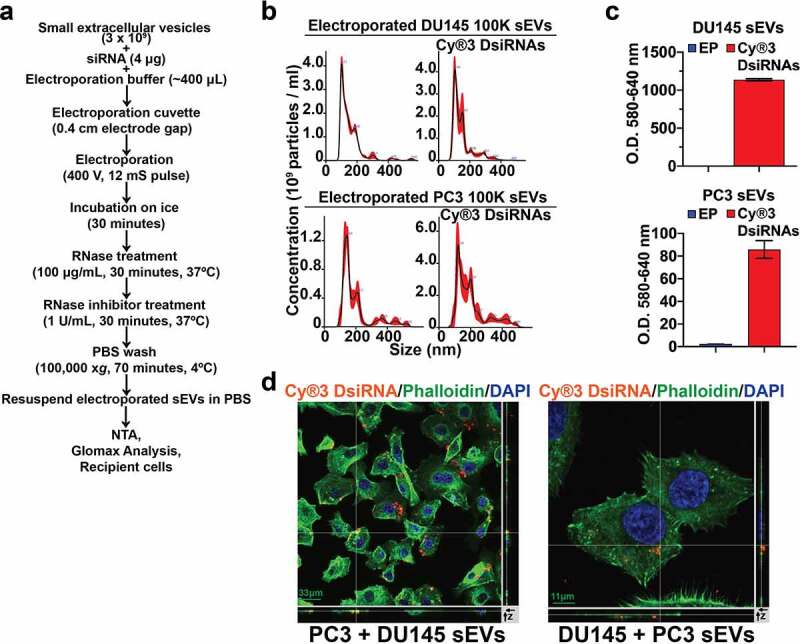
**Small Extracellular Vesicle (sEV)-mediated delivery of Cy®3 DsiRNAs to prostate cancer cells**. (a) Schematic representation of the experimental approach for siRNA electroporation into sEVs followed by different analyses. (b) The upper panel shows NTA of the DU145 sEVs electroporated without DsiRNAs or DU145 sEVs electroporated with Cy®3 DsiRNAs. The bottom panel shows NTA of PC3 sEVs electroporated without DsiRNAs or PC3 sEVs electroporated with Cy®3 DsiRNAs. (c) Fluorescence intensity from DU145 sEVs electroporated without DsiRNAs (EP) or DU145 sEVs electroporated with Cy®3 DsiRNAs (top panel) and PC3 sEVs electroporated without DsiRNAs (EP) or PC3 sEVs electroporated with Cy®3 DsiRNAs (bottom panel). Bar graphs show fluorescence measured as Optical Density (O.D.) in arbitrary units (a.u.) at 580–640 nm. Values are presented as mean ± SEM. (d) Confocal microscopy analysis was carried out to evaluate internalization of sEVs loaded with Cy®3 DsiRNAs into PrCa cells. Left, Z-stack analysis showing DU145-derived sEV-mediated internalization of Cy®3 DsiRNAs into PC3 recipient cells. Right, Z-stack analysis showing PC3-derived sEV-mediated internalization of Cy®3 DsiRNAs into DU145 recipient cells. Cy®3 DsiRNA emits Orange fluorescent signal, FITC Phalloidin was used to label actin (green) and DAPI was used to detect nuclei (blue). Left, scale bar = 33 µm; right, scale bar = 11 µm.

With the objective to utilize sEVs for delivery of siRNAs into PrCa cells, we adapted an experimental protocol published previously,^34^ and optimized it to electroporate DU145 and PC3 sEVs with siRNAs (Figure 3a). To visualize the sEV-mediated delivery of siRNAs into PrCa cells, we utilized fluorescently-labeled Cy®3 DsiRNAs for loading into sEVs. , Since electroporation can cause aggregation and voltage-mediated damage of EVs^46,47^ , we first investigated whether electroporation-mediated loading of DU145 and PC3 sEVs with Cy®3 DsiRNAs impact the size of sEVs. Our NTA data show that the majority of the DU145 or PC3 sEVs pre- (data not shown) or post-electroporation without or with Cy®3labeled DsiRNAs exhibit a comparable particle size of <150 nm (Figure 3b). To investigate the efficiency of Cy®3 DsiRNA loading into DU145 and PC3 sEVs, we measured the fluorescence of Cy®3 DsiRNAs encapsulated within sEVs with excitation at 520 nm and emission at 580–640 nm. A high Cy®3 fluorescence from DU145 and PC3 sEVs loaded with Cy®3 DsiRNAs is detected compared to DU145 and PC3 sEVs loaded without Cy®3 DsiRNAs (Figure 3c). We further investigated whether DU145 and PC3 sEVs encapsulating Cy®3 DsiRNAs are efficiently internalized into the PrCa cells. For this experiment, we incubated PC3 recipient cells with DU145-derived sEVs loaded with Cy®3 DsiRNAs, and DU145 recipient cells with PC3-derived sEVs loaded with Cy®3 DsiRNAs, and performed confocal microscopy. Z-stack analysis of confocal microscopy images reveals that Cy®3 DsiRNAs-loaded DU145 and PC3 sEVs are internalized and colocalize with endogenous actin in PC3 (28.7%) and DU145 (27.4%) recipient cells (Figure 3d).

### Small extracellular vesicle-mediated transfer of *ITGB6*-targeting siRNAs to prostate cancer cells inhibit adhesion and migration on LAP-TGFβ1.

**Figure 4.**
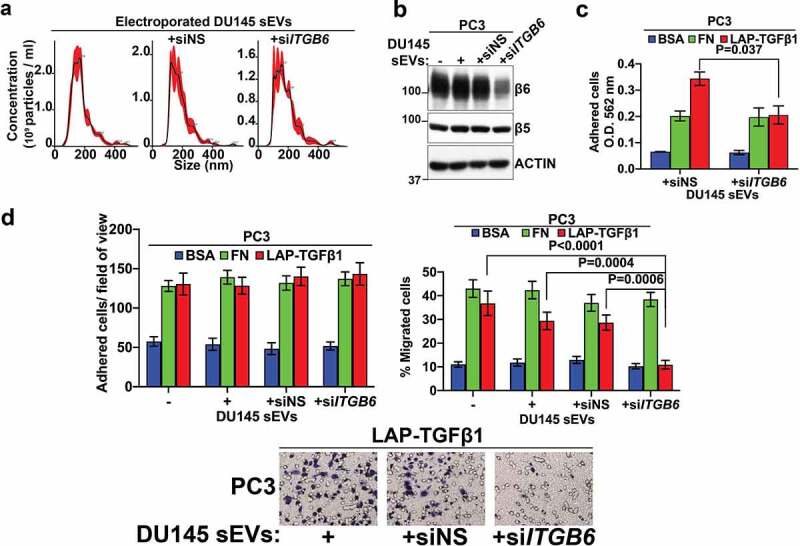
***In vitro* efficacy of small Extracellular Vesicles (sEVs) loaded with siRNAs targeting *ITGB6***. (a) NTA of the density gradient-isolated DU145 sEVs electroporated without siRNAs (left), electroporated with non-silencing siRNAs (+siNS, middle), or electroporated with siRNAs targeting *ITGB6* (+si*ITGB6*, right). (b) IB analysis of TCL from PC3 parental cells (-), PC3 cells treated with DU145 sEVs electroporated without siRNAs (+), +siNS sEVs, or +si*ITGB6* sEVs for expression of β6 subunit, β5 subunit, and ACTIN as loading control. (c) PC3 cells treated with +siNS, or +si DU145 sEVs were seeded (2.5x10^4^, 3 replicates) for 2.5 hours on BSA (1%), fibronectin (FN, 10 µg/mL), or LAP-TGFβ1 (10 µg/mL)-coated wells. The bar graphs represent the degree of cell adhesion quantified as O.D. of crystal violet staining measured at 562 nm. The values are presented as mean ± SEM; P values were calculated by the two-group t-test with Welch’s correction. (d) The PC3 parental cells (-), PC3 cells treated with DU145 sEVs electroporated without siRNAs (+), +siNS sEVs, or +si*ITGB6* sEVs were plated (5x10^4^, 3 replicates) on BSA (1%), FN (10 µg/ml), LAP-TGFβ1 (7 µg/mL)-coated Transwell chambers in serum-free media in the top and bottom chambers. The bar graphs in the left panel represent the adhered PC3 cells/field of view (FOV) in 6 hours on BSA, FN, or LAP-TGFβ1 in each treatment group (15 fields for each condition; FOV = 0.044 mm diameter). The bar graphs in the right panel represent the percentage of PC3 cells migrated in 6 hours on BSA, FN, or LAP-TGFβ1 toward the bottom chambers in each treatment group (24 fields for each condition; FOV = 0.044 mm diameter). The values are reported as mean ± SEM; The P values were calculated by ANOVA with post-hoc Holm-Sidak’s multiple comparisons test for migration on BSA and FN, and the Kruskal-Wallis test with post-hoc Dunn’s multiple comparisons test for migration on LAP-TGFβ1. The lower panels show representative images of PC3 cells migrated on LAP-TGFβ1 toward the bottom of Transwell chambers upon treatment with DU145 sEVs electroporated without siRNAs (+), +siNS sEVs, or +si*ITGB6* sEVs.

We hypothesize that sEV-mediated delivery of *ITGB6*-targeting siRNAs into αVβ6 integrin expressing PC3 cells might inhibit expression of the αVβ6 integrin and confer an integrin loss-of-function phenotype. Our group has previously shown that the β6 subunit is expressed in PrCa cell-derived sEVs and efficiently transferred, via these sEVs, to other prostate cells.^6^ Therefore, to avoid the possibility of sEV-mediated transfer of β6 subunit to recipient cells, we utilized DU145 sEVs (which do not express the β6 subunit) for electroporation-mediated loading with *ITGB6*-targeting siRNAs. Our NTA data reveal comparable sizes of DU145 sEVs electroporated without siRNAs, with non-silencing siRNAs (+siNS sEVs), or with D1, an *ITGB6*-targeting siRNA (+si*ITGB6* sEVs) (Figure 4a). IB analysis shows a reduction in expression of the β6 subunit but no change in expression of the β5 subunit, which is also known to associate with the αV integrin subunit, in TCL from PC3 cells treated with +si*ITGB6* sEVs compared to PC3 parental cells (-), PC3 cells treated with sEVs electroporated without siRNAs (+), or +siNS sEVs (Figure 4b). In a separate experiment, we also tested a different human *ITGB6-*targeting siRNA (D13.1). D13.1 siRNA effectively downregulates β6 subunit only upon oligofectamine-mediated transfection of PC3 cells (Supplementary Figure 1a) but not without oligofectamine (Supplementary Figure 1b). Treatment of PC3 cells with DU145-derived sEVs loaded with D13.1 effectively reduces expression of β6 compared to PC3 parental cells (-), PC3 cells treated with sEVs electroporated without siRNAs (+), or +siNS sEVs (Supplementary Figure 1c).

**Figure 5.**
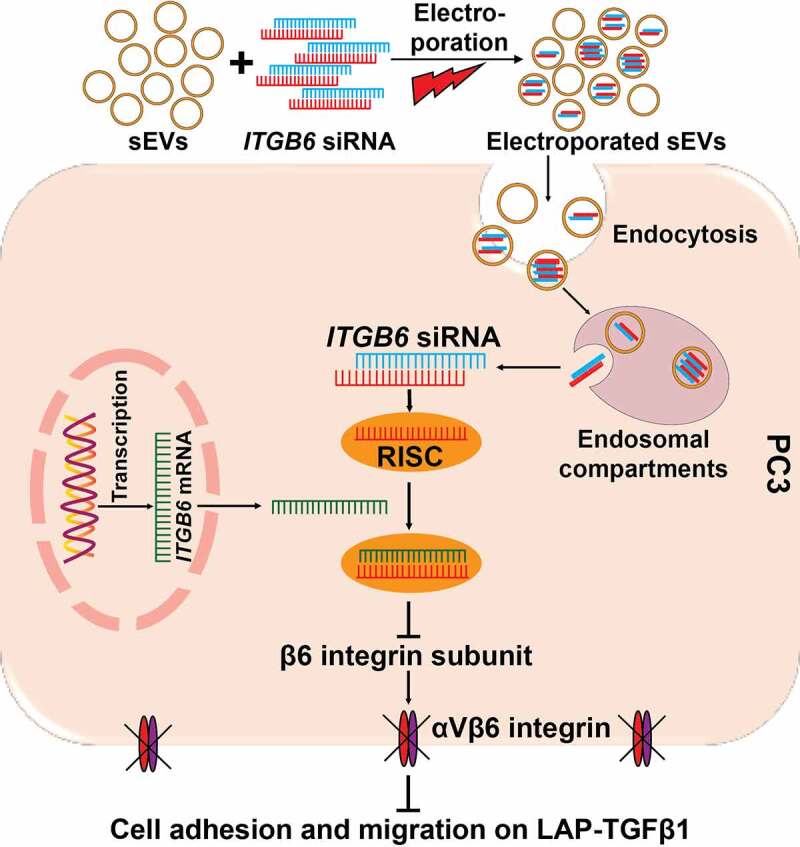
**Proposed model for small Extracellular Vesicle (sEV)-mediated delivery of *ITGB6* targeting siRNAs in AR-negative prostate cancer cells**. The schematic diagram shows that DU145 sEVs electroporated with *ITGB6* siRNAs, upon internalization into PC3 cells, release the cargo into the cytoplasm out of the endosomal compartment; this results in RISC-mediated silencing of *ITGB6* mRNA and consequent reduced expression of the β6 subunit. This reduced expression of the β6 subunit in turn leads to reduced adhesion and migration of PC3 cells on the αVβ6-specific ligand LAP-TGFβ1.

We next investigated whether inhibition of the β6 subunit in PC3 cells by +si*ITGB6* sEVs might affect the ability of PC3 cells to adhere and migrate on extracellular matrix proteins such as fibronectin (FN) and LAP-TGFβ1, two major ligands found in the tumor microenvironment and specific for the αVβ6 integrin.^9,48^ We find that upon inhibition of the β6 subunit by +si*ITGB6* sEVs, there is no significant change in PC3 cell adhesion and migration on FN (Figure 4c, d) compared to PC3 cells that were treated with +siNS sEVs. In contrast, inhibition of the β6 subunit due to +si*ITGB6* sEVs significantly abrogates adhesion of PC3 cells on LAP-TGFβ1 compared to PC3 cells that were treated with +siNS sEVs (P = .037, Figure 4c). Furthermore, treatment with +si*ITGB6* sEVs of PC3 cells that do adhere under longer incubation conditions on LAP-TGFβ1-coated Transwell inserts (Figure 4d, left panel) shows no effect on cell attachment, but a significant reduction in migration on LAP-TGFβ1 compared to parental cells (-) (P < .0001) as well as PC3 cells treated with sEVs electroporated without siRNAs (+) (P = .0004) or +siNS sEVs (P = .0006) (Figure 4d, right& bottom panel). Overall, our data indicate that DU145-derived sEV-mediated delivery of *ITGB6*-targeting siRNAs into PC3 recipient cells not only efficiently downregulates expression of the β6 subunit in these cells, but also confers as scratchedadhesive and migratory phenotypes that closely resemble those caused by the loss of αVβ6 integrin functions (Figure 5).

## Discussion

Here, we show for the first time that expression of the *ITGB6* transcript and expression of the αVβ6 integrin protein have a significant negative correlation with AR expression in mCRPrCa patient tumors and LuCaP PrCa PDX models, respectively. Expression of AR in an AR-deficient cell line results in downregulation of the β6 integrin subunit. Furthermore, by sEV-mediated delivery of *ITGB6*-targeting siRNAs into PrCa cells, we demonstrate a significant abrogation of the β6 subunit expression and consequent inhibition of adhesive and migratory potential of the recipient PrCa cells. Overall, our data suggest that sEV-mediated delivery of *ITGB6*-targeting siRNAs could serve as a potential therapeutic modality against the αVβ6 integrin-positive PrCa tumors.

According to published reports, the expression pattern of the αVβ6 integrin is highly heterogeneous in primary prostate tumors; it is highly expressed in bone metastatic PrCa cases, whereas its expression is undetectable in neuroendocrine PrCa (NEPrCa) cases and normal prostatic epithelium.^23,24,43^ However, none of these studies examined the correlation between the αVβ6 integrin and AR expression in PrCa patients. *In vitro*, we show that the β6 subunit is not expressed in AR+ PrCa cells (C4-2B, LNCaP) whereas it is expressed in AR- PrCa cells (PC3, NCI-H660). Notably, our data reveal that β6 expression is absent in AR- DU145 cells; we speculate that this observation could be attributed to DNA methylation, post-transcriptional silencing of *ITGB6*, or post-translational silencing of the αVβ6 integrin in DU145 cells. Taking into consideration our previous and current findings, we propose the emergence of AR+, αVβ6- and AR-, αVβ6+ tumor subtypes in addition to previously described AR+, αVβ6+ subtype during PrCa progression.^23,41^ Co-existence of these three subtypes within the same prostate tumor could also be a possibility. Both AR and the αVβ6 integrin are expressed in PrCa cell-derived sEVs;^6,49^ and the sEV-mediated transfer of these molecules between prostate tumor subtypes as well as its impact on PrCa progression need further investigation. Thus, increased expression of the αVβ6 integrin during PrCa progression and its oncogenic manifestations,^6,7,23,24,26,29^ makes it an attractive target for the αVβ6+ subsets in PrCa.

Previous attempts to target the αVβ6 integrin using BG00011 antibody have raised issues related to safety and efficacy.^50^ Additionally, the strategies to therapeutically target the αVβ6 integrin in different cancers have not yet as scratcheden successful.^51^ As an alternative strategy, significant interest has emerged in utilizing sEVs as potential tumor-targeted vehicles for cancer therapy. sEVs provide a relatively stable environment for transport of the therapeutic agent of choice, can be modified to improve cell-specific targeting, and have the ability to enter the cells, thus allowing the therapeutic cargo to be delivered.^33,39,52,53^ With regard to toxicity and immunogenicity as main challenges of cell-based therapies, sEVs are thought to be well tolerated and deliver cargo with minimal immune clearance.^33^ Multiple studies have reported successful utilization of electroporation as a technique to load siRNAs into sEVs and downregulate the expression of specific targets.^32,34,40,54^ A few studies have also reported that electroporation can induce aggregation and at high voltages cause damage of EVs.^46,47^ Our NTA data show a comparable pre- and post-electroporation sEV sizes suggesting that electroporation of siRNAs into sEVs does not cause aggregation or voltage-induced damage to sEVs in our system. Furthermore, to investigate the internalization efficiency of sEVs encapsulating siRNAs into the PrCa cells, we utilized fluorescently-labeled Cy®3 DsiRNAs. Consistent with previous findings,^32,55–58^ we observe an efficient internalization of sEVs loaded with Cy®3 DsiRNAs. Overall, our data suggest that electroporation-mediated encapsulation of siRNAs into sEVs does not alter sEV integrity or internalization efficacy.

Our data for the first time demonstrate that sEVs encapsulating *ITGB6-targeting* siRNAs abrogate expression of the β6 subunit in PrCa cells. In terms of specificity, no change in the levels of the β5 subunit, which is also known to associate with the αV integrin subunit, was observed, thus indicating that there are no apparent off-target effects of sEV-mediated *ITGB6* siRNA delivery in PrCa cells. It has been recently reported that within recipient cells, the EVs are internalized in endo/lysosomal compartments; the mechanisms associated with endo/lysosomal escape of siRNA cargo remain unknown.^59,60^ The efficient inhibition of the β6 subunit in PrCa cells indicates that upon sEV-mediated internalization, the *ITGB6* siRNAs could bypass degradation by the endo/lysosomal pathway. We observe a significantly reduced adhesion and migration of PC3 cells treated with *ITGB6* siRNA-loaded sEVs on the αVβ6-specific substrate, LAP-TGFβ1. However, we do not observe any significant impact on adhesion or migration of these cells on fibronectin. This result could be attributed to the involvement of another fibronectin receptor such as the β1 integrin^48^ that is abundantly expressed by these PrCa cells. In this study, for *ITGB6* siRNA delivery, we utilized sEVs derived from DU145 PrCa cells that do not express the β6 subunit. However, using PrCa cell-derived sEVs for preclinical and clinical intervention of the αVβ6 integrin and consequent reduction in prostate tumor growth and metastases, may pose challenges. In this context, it has been demonstrated that sEVs isolated from bone marrow-derived mesenchymal stem cells (MSC-sEVs) are stable in circulation due to the presentation of CD47 on their surface.^35^ Therefore, future *in vitro* and *in vivo* studies aiming at inhibiting of the αVβ6 integrin in prostate tumors could potentially be optimized by using MSC-sEVs.

Collectively, our results indicate that by employing sEV-mediated delivery of siRNAs targeting the *ITGB6* gene, the functional abrogation of the αVβ6 integrin might be achieved in human PrCa cells. Further evaluation of this sEV-mediated targeting of prostate tumors in preclinical and clinical models might ultimately lead to the development of new therapeutic methods to prevent and/or delay prostate cancer progression to advanced stages.

## Materials and methods

### RNA-sequencing of mCRPrCa samples

RNA-sequencing analysis of mCRPrCa specimens acquired through rapid autopsy was performed, as previously described.^41^ Based on expression of the *AR* transcript, the mCRPrCa specimens were classified as *AR*+ (n = 89) or *AR*- (n = 19). *ITGB6* Log_2_ Fragments Per Kilobase of transcript per Million (FPKM) mapped reads in these *AR*+ and AR- mCRPrCa specimens were compared, as described in the statistical analysis section below.

### LuCaP PDX tumor microarray (TMA) IHC assessment

The TMA containing 42 LuCaP PDX models were utilized for the IHC staining of the αVβ6 integrin followed by generation of an IHC score, as previously published.^43^ Based on their IHC staining scores for AR expression, these 42 LuCaP PDXs were classified as AR+ (n = 36) or AR – (n = 6). The IHC scores for the αVβ6 integrin in AR+ and AR- LuCaP PDXs were compared, as described in the statistical analysis section below.

### Prostate cancer transcriptome atlas (PCTA)

The transcriptome data of mCRPrCa cases (n = 260) were downloaded from the PCTA web tool.^44^ The association between transcript levels of *ITGB6* and *AR* was analyzed as described in the statistical analysis section below.

### Cell lines

All PrCa cells (C4-2B, DU145, LNCaP, NCI-H660, PC3) were purchased from ATCC, and cultured in a humidified atmosphere of 5% CO_2_ at 37°C using their respective medium. The C4-2B and LNCaP cells were cultured in RPMI medium (Corning, 10–040-CV) supplemented with 5% fetal bovine serum [(FBS), Hyclone, SH30396.03], 1 mM sodium pyruvate (Corning, 25–000-CI), 5 mL of MEM non-essential amino acids (Corning, 25–025-CI), 50 U/mL penicillin and 50 μg/mL streptomycin [(PenStrep), Corning Cellgro, 30–001-CI)]. The DU145 cells were cultured in DMEM medium (Corning, 10–013-CV) supplemented with 10% FBS, and PenStrep. The NCI-H660 cells were cultured in RPMI medium supplemented with 5% FBS, 5 mL of ITS liquid medium supplement (Sigma-Aldrich, 13146), 10 nM Hydrocortisone (Sigma-Aldrich, H0135), 10 nM beta-estradiol (Sigma-Aldrich, E227), 5 mL of L-glutamine (Gibco, 25030081), and PenStrep. The PC3 cells were cultured in RPMI media supplemented with 10% FBS and PenStrep.

### Transient expression of AR-V7 and AR-WT

For transient expression of AR, PC3 cells were plated (2x10^5^) in a 6-well plate and grown overnight at 37°C. The following day, the cells were washed with phosphate buffered saline (PBS) and incubated with 1 mL serum-free medium at 37°C for 2 hours. For transfection, 4 μg pEGFP-C3 empty vector, pEGFP-C1-AR-V7 vector or pEGFP-C1-AR-WT vector were mixed with 12 μL of lipofectamine 2000 (Invitrogen, 11668–019) in 200 μL of serum-free RPMI medium. The pEGFP-C3-lipofectamine, pEFGP-C1-AR-V7-lipofectamine, or pEFGP-C1-AR-WT-lipofectamine mix were incubated at room temperature for 25 minutes and added drop-wise to PC3 cells followed by incubation at 37°C for 6 hours. After 6 hours, 700 μL of complete medium (without PenStrep) were added to the cells and incubated at 37°C, overnight. The following day, a second round of transfection was performed as described above and after overnight incubation at 37°C, the transfection medium was replaced with complete medium and the cells were incubated at 37°C for 48 hours followed by preparation of TCL as mentioned below.

### siRNA transfection of PC3 PrCa cells

Using oligofectamine, PC3 parental cells were transiently transfected with 100 nM of non-silencing (NS) siRNA (Cat. No. D-001810-01-20; Dharmacon), human *ITGB6* targeting siRNA duplex D1 (sense: 5’-AGGACTCAACTTGUCATTTACAGCC-3’, antisense: 5’-GGCUGUAAAUGACAAGUU-3’) or D13.1 (sense 5’ GUCACUUGGACAGCAAGAAUGAATA-3’, antisense 3’-GACAGUGAACCUGUCGUUCUUACUUAU-5’), as previously described.^61^   PC3 parental cells were also treated with 100 nM of NS or D13.1 siRNAs without oligofectamine. Briefly, 2x10^5^ cells were plated in a 6-well plate for 24 hours. Then, cells were serum starved and 100 nM of NS or D13.1 siRNA without oligofectamine were added to the respective wells dropwise. Cells were incubated at 37°C for 8 hours. After 8 hours, complete medium was added to the cells. The same process was repeated the following day. After two rounds of siRNA treatment cells were lysed.

### Immunoblotting (IB) analyses and antibodies

For IB, total cell lysates (TCL) or sEV lysates were prepared using Radio Immuno Precipitation Assay (RIPA) buffer (10 mM Tris-HCl, pH 7.4, 150 mM NaCl, 1 mM EDTA, 0.1% SDS, 1% Triton X-100, and 1% sodium deoxycholate) supplemented with protease inhibitors (calpain, aprotinin, leupeptin, pepstatin, sodium fluoride, sodium orthovanadate). The total protein concentration of lysates was determined using *DC*^TM^ protein assay kit (Bio-Rad, 5000112) as per the manufacturer’s protocol. Equal amounts of proteins in non-reducing (heated without 2-Mercaptoethanol) and reducing conditions (heated with 2-Mercaptoethanol) were separated by Sodium Dodecyl Sulfate-Polyacrylamide Gel Electrophoresis (SDS-PAGE), transferred to polyvinylidene difluoride (PVDF) membranes (Millipore, Immobilon-E PVDF membrane, pore size 0.45 µm, IEVH00005), blocked with buffer (5% nonfat dry milk) in Tris Buffer Saline with 0.1% Tween 20 (TBST) at room temperature for 1 hour, incubated overnight with primary Abs at 4°C, as described below, followed by TBST washes at room temperature (4 x 10 minutes), incubation with horseradish peroxidase (HRP)-conjugated anti-goat, -mouse or -rabbit secondary Abs as described below for 1 hour at room temperature, followed by TBST washes (4 x 10 minutes) at room temperature. For visualization, WesternBright^TM^ ECL HRP substrate kit (Advansta, K-12045-D50) was used.

The following primary antibodies (Abs) were used for IB analyses: goat polyclonal Ab against the αVβ6 integrin (R&D Systems, AF2389); mouse monoclonal Abs against: αVβ6 integrin (Biogen, 6.2A1), ALIX (Abcam, ab117600), AR (Santa Cruz, sc-7305), CD9 (Santa Cruz, sc-13118), CD63 (Abcam, ab8219), CD81 (Abcam, ab23505); rabbit monoclonal Abs against β5 integrin subunit (Cell Signaling, 3629), TSG101 (Abcam, ab125011); rabbit polyclonal Abs against: ACTIN (Sigma Aldrich, A2066), Calnexin (CANX) (Cell Signaling, 2433S), and rabbit antiserum against αV subunit (C-terminus). The following secondary Abs were used for IB analyses: HRP-linked anti-goat IgG (R&D Systems, HAF019), HRP-linked anti-mouse IgG (Cell Signaling, 7076S) and HRP-linked anti-rabbit IgG (Cell Signaling, 7074S).

### Small extracellular vesicle isolation

As published previously,^62^ for sEV isolation, PrCa cells (PC3 and DU145) were plated in 150 mm cell culture dishes (ThermoScientific, 130183) in their respective complete medium, as described above. After 48 hours of incubation at 37°C, cells were washed with PBS and incubated with serum-free medium (complete media devoid of FBS) for the next 48 hours. The sEVs were isolated by high-speed differential ultracentrifugation of the supernatant (SN) collected after 48 hours of serum-starvation. The dead cells and cell debris were spun down from SN at 2000 *xg*, 4°C for 20 minutes. The SN collected was spun at 10,000 *xg*, 4°C for 35 minutes. Next, the SN collected without disturbing the 10,000 *xg* pellet was spun at 100,000 *xg*, 4°C for 70 minutes; the pellet was washed in 40 mL PBS followed by a second spin at 100,000 *xg*, 4°C for 70 minutes. The 10,000 *xg* and 100,000 *xg* centrifugation were done in a Beckman Type 45Ti rotor using a Beckman L8-70M  Ultracentrifuge. The final sEV pellets were resuspended in 100 μL PBS.

### Small extracellular vesicle isolation by iodixanol density gradient centrifugation

For iodixanol density gradient separation, the sEVs obtained from PC3 or DU145 cells were suspended in 1.636 mL of 30% iodixanol-buffer solution [made by mixing 1:1 of 60% (weight/volume) stock solution of iodixanol (Sigma, OptiPrep^TM^, D1556) with a stock buffer [(0.25 M sucrose, 10 mM Tris (pH 8.0), 1 mM EDTA, pH 7.4)]    and layered at the bottom of an ultracentrifugation tube. Next, 0.709 mL of 20% iodixanol-buffer solution and 0.654 mL of 10% iodixanol-buffer solution were successively layered on top of the 30% iodixanol-vesicle suspension to create a discontinuous gradient. The gradient samples were centrifuged for 70 minutes at 350,000 *xg*, 4°C in a Sorvall TST 60.4 swinging bucket rotor using a Beckman L8-70M  Ultracentrifuge. Ten consecutive fractions of 0.267 mL were collected from top to bottom of the gradient. The refractive index of each fraction was assessed with an ABBE-3L  refractometer (Fisher Scientific) to calculate the density of each fraction. All ten fractions were diluted with 1 mL PBS and centrifuged for 70 minutes at 100,000 *xg*, 4°C in a S55A2 rotor using a Sorvall^TM^ MTX 150 Micro-Ultracentrifuge. The pellets from the first five sEV fractions (F1-F5) were pooled and washed in 1 mL PBS and centrifuged for 70 minutes at 100,000 *xg*, 4°C in a S55A2 rotor using a Sorvall^TM^ MTX 150 Micro-Ultracentrifuge. The final pellet was resuspended in 100 μL of PBS and stored at −80°C or utilized for analysis by NTA, IB, or electroporation as described below.

### Electroporation of small extracellular vesicles with siRNAs

For electroporation, DU145 or PC3 sEVs (3 x 10^9^) were mixed with 4 μg of non-silencing siRNAs (Dharmacon, D-001810-01-20), *ITGB6* targeting D1 or D13.1 siRNA duplex, or Cy®3 transfection control DsiRNAs (Integrated DNA Technologies, 51–01-03-08) in ~400 μL of cold electroporation buffer (1.15 mM K_2_HPO_4_ pH 7.2, 25 mM KCl, 21% Optiprep), and transferred to ice cold electroporation cuvettes (Bio-Rad Gene Pulser® Cuvette, 165–2088). sEV electroporation was performed using Bio-Rad MicroPulsar^TM^ Electroporation Apparatus (165–2100) using a 12 milli-second pulse (6 pulses of 2 milli-second each) at 400 volts. After electroporation, sEVs were incubated on ice for 30 minutes followed by incubation with 100 μg/mL RNase A (ThermoFisher Scientific, EN0531) at 37°C for 30 minutes. This was followed by incubation with 1 U/mL of RNase inhibitor (Invitrogen from ThermoFisher Scientific, AM2694) at 37°C for 30 minutes. The sEVs were washed with 3 mL PBS and centrifuged using a TLA100.3 rotor in a Beckman Optima^TM^ TL Ultracentrifuge at 100,000 *xg* for 70 minutes, washed with 1 mL PBS and centrifuged using a S55A2 rotor in a SorvallTM MTX 150 Micro-Ultracentrifuge at 100,000 *xg* for 70 minutes, or washed with 30 mL PBS and centrifuged using a Beckman Type 45 Ti rotor in a L7-65 Ultracentrifuge at 100,000 *xg* for 70 minutes. After centrifugation, the PBS was removed and the respective sEV pellets electroporated with siRNAs were pooled and resuspended in 100 μL PBS per sample.

The DU145 and PC3 sEVs electroporated with Cy®3 DsiRNAs were resuspended in 100 μL PBS, plated in black 96-well plates (Thermo Scientific, 137101) and absorbance of Cy®3 fluorescence was measured using the filter (Ex: 520 nm, Em: 580–640 nm) on a GloMax® Discover system (Promega Corporation, USA).

### Nanoparticle tracking analysis (NTA)

The size distribution and concentration of sEVs isolated from the PrCa cells (PC3 and DU145) pre- and post-electroporation with Cy®3 DsiRNAs, non-silencing siRNAs, or *ITGB6*-targeting siRNAs were analyzed using a NanoSight NS300 instrument (Malvern, UK). Briefly, sEV suspensions were diluted 1:1000 and/or 1:200 (F1-F5 sEVs) in PBS, and the analysis was performed using camera settings ranging from 11–13 to visualize the sEV particles. Using the script SOP standard measurement, video files of 30-second duration (repeated three times) were captured at a frame rate of 25 frames per second of particles moving under Brownian motion at a temperature ranging from 22–25°C. The analyses of the videos were performed at a detection threshold of 5 using NTA software version 3.1 (build 3.1.54).

### Immunofluorescence and confocal microscopy

PC3 and DU145 cells (10^4^) were cultured on fibronectin-coated (10 μg/mL) glass coverslips (Fisher Scientific, 12–545-100) in 12-well cell culture dishes for 48 hours and incubated with DU145- or PC3-derived sEVs electroporated with Cy®3 DsiRNA or PBS for 18 hours. Subsequently, cells were washed with PBS (2 x 5 minutes) at room temperature, fixed with 4% paraformaldehyde (PFA) for 15 minutes at room temperature, and washed with PBS (5 minutes x 3 washes) at room temperature. Cells were quenched with 50 mM ammonium chloride for 15 minutes at room temperature and washed with PBS (2 x 5 minutes) at room temperature. Cells were then permeabilized with 0.25% Triton-X 100 for 10 minutes at room temperature, washed with PBS (3 x 5 minutes) at room temperature, and blocked with 5% bovine serum albumin in PBS with 0.1% Tween 20 (PBST) for one hour at room temperature. Cells were then incubated with Fluorescein isothiocyanate labeled-phalloidin (5 μg/mL, Sigma-Aldrich, P5282) at room temperature for 1 hour, and washed with PBST (3 x 5 minutes) at room temperature. Glass cover slips were then mounted on VWR vistavision^TM^ microscope glass slides (VWR International, 16004–422) using ProLong™ diamond antifade mountant with DAPI (Invitrogen, P36966). The slides were analyzed and images were captured using a Nikon A1R confocal microscope. To evaluate internalization of Cy®3 DsiRNA electroporated sEVs into PC3 and DU145 cells, Z-stack image analysis was performed using NIS Elements Viewer (version 4.11.0) imaging software. The number of PC3 cells counted was 198 and of DU145 cells was 102.

### Inhibition of αVβ6 integrin expression using small extracellular vesicles electroporated with siRNAs

PC3 cells (2.5 x 10^5^) were plated in a 6-well cell culture dish using 2 mL of complete media as mentioned above and kept in a 37°C cell culture incubator. The following day, PC3 cells were washed with PBS, supplemented with 1 mL of serum-free media, and treated with iodixanol density gradient purified DU145 sEVs electroporated without siRNAs (+), DU145 sEVs electroporated with non-silencing siRNAs (+siNS sEVs), or *ITGB6*-targeting siRNAs (+si*ITGB6* sEVs) and incubated overnight at 37°C. The following day, PC3 cells were treated with a second round of electroporated DU145 sEVs as mentioned above, and incubated overnight at 37°C. The following day, PC3 cells treated with the respective sEVs were washed with PBS, and incubated with 2 mL of complete media at 37°C. After 7.5 hours, PC3 cells were washed with PBS followed by cell lysis.

### Cell adhesion assay

For cell adhesion assays, 96-well flat bottom adhesion assay plates (Linbro®/Titertek®, 76–232-05) were coated with 150 μL or 200 μL of 1% BSA in PBS or fibronectin (10 μg/ml in PBS) or recombinant human LAP-TGFβ1 (10 μg/ml in PBS, R&D, 246-LP) overnight at 4°C. On the day of the assay, the coated wells were blocked with 1% BSA in PBS for 1 hour at room temperature followed by washing with PBS three times. PC3 cells treated with DU145 sEVs electroporated with non-silencing siRNAs (+siNS sEVs) or *ITGB6*-targeting siRNAs (+si*ITGB6* sEVs) were washed with PBS, detached using 0.05% trypsin-EDTA (Corning, 25–052-CI) and rinsed with 1 mg/mL soybean trypsin inhibitor (Roche, 10109886001). Cells were spun down, re-suspended in serum-free media, counted by hemocytometer, plated (2.5 x 10^4^/ 200 μL , 3 replicates) on coated wells and allowed to settle at 37°C. After 2.5 hours, media were gently aspirated from wells and cells were washed with PBS (three washes) to remove the non-adhered cells. For fixing, adhered PC3 cells were incubated with 200 μL of 3% PFA in each well for 30 minutes at room temperature. After fixation, cells were washed with PBS (three washes) and stained with crystal violet (0.5% in water) for 3 hours or overnight at room temperature. The excess crystal violet was gently washed with tap water and wells were air-dried. The optical density (O.D.) of stained PC3 cells were measured at 562 nm in a spectrophotometer (ThermoScientific Multiskan Spectrum) using ScanIt software 2.4.4.

### Transwell migration assay

For Transwell migration assays, the upper and bottom membranes of Transwell inserts (12 μm pore size, Millicell, PIXP01250) were coated the day before the assay, with 1% BSA, 10 μg/mL of Fibronectin, or ~7 μg/mL of recombinant human LAP-TGFβ1 (R&D systems, 246-LP), overnight at 4°C. The following day, inserts were washed once with PBS, blocked with 1% BSA for 30 minutes at room temperature, followed by two PBS washes. PC3 parental cells, PC3 cells treated with DU145 sEVs electroporated without siRNAs (+), DU145 sEVs electroporated with NS siRNAs (+siNS sEVs), or *ITGB6-targeting* siRNAs (+si*ITGB6* sEVs) were trypsinized, neutralized with soybean trypsin inhibitor (1 mg/mL), counted using a hemocytometer, and plated (5 x 10^4^) using 300 μL serum-free media in Transwell inserts (3 replicates). The bottom chamber of Transwell inserts also consisted of 300 μL serum-free media. Cells were then incubated at 37°C for 6 hours. Media were then removed from top and bottom of inserts followed by three washes with PBS. Cells on the top and bottom of the Transwell inserts were fixed using 3% PFA for 30 minutes at room temperature. PFA was removed from the top and bottom of inserts by two PBS washes. Cells on the top and bottom of inserts were stained with crystal violet solution (0.5% crystal violet in water) for 30 minutes at room temperature. Extra crystal violet stain was washed with water and inserts were dried. Photomicrographs of the top of each Transwell insert indicating total number of cells [5 different field of view (FOV), FOV = 0.044 mm diameter] were captured under an inverted microscope (Nikon Eclipse TS100). Using a cotton-tipped applicator, cells from the top of the Transwell inserts were removed and images of cells migrated to the bottom of the Transwell insert (8 different FOV, FOV = 0.044 mm diameter) were captured under the inverted microscope. The number of cells in each FOV mentioned above were counted manually. The mean of percentage of migrated cells [(cells migrated to bottom/total number of cells) x 100] in each condition was calculated and plotted as a bar graph.

### Statistical analyses

*ITGB6* Log_2_ FPKM expression values from RNA-sequencing of mCRPrCa cases classified as AR- and AR+ were compared using the two-tail Mann-Whitney non-parametric test. The αVβ6 integrin IHC scores in LuCaPs grouped as AR- and AR+ were converted to Log_2_(1+ IHC score) and compared using the two-tail Mann-Whitney non-parametric test. The GraphPad Prism was used to generate bar graphs.

Data for *ITGB6* and *AR* transcript levels in mCRPrCa cases were downloaded from the PCTA and the association between *ITGB6* and *AR* expression was tested using the Spearman correlation test to generate rho and P-value using the *corr*. test R function. *ggplot2* was used to generate scatter plots with linear regression lines.

The two-group t-test with Welch’s correction was used to compare the mean of adhered PC3 cells between groups. ANOVA with post-hoc Holm-Sidak’s multiple comparison test or the Kruskal-Wallis test with post-hoc Dunn’s multiple comparisons test was used to compare the number of cells per FOV that adhered to Transwell inserts between groups. ANOVA with post-hoc Holm-Sidak’s multiple comparison test was used to compare the mean of % migrated cells between groups on BSA and FN. The Kruskal-Wallis test with post-hoc Dunn’s multiple comparisons test was used to compare the mean of % migrated cells between groups on LAP-TGFβ1. GraphPad Prism was used to generate bar graphs.

## Supplementary Material

Supplemental Material
