# Supplementary figures and images for: Small extracellular vesicle-mediated ITGB6 siRNA delivery downregulates the αVβ6 integrin and inhibits adhesion and migration of recipient prostate cancer cells
Source: Cancer Biol Ther. 2022 Feb 19;23(1):173–85. doi: 10.1080/15384047.2022.2030622 (PMC8865252; doi:10.1080/15384047.2022.2030622)

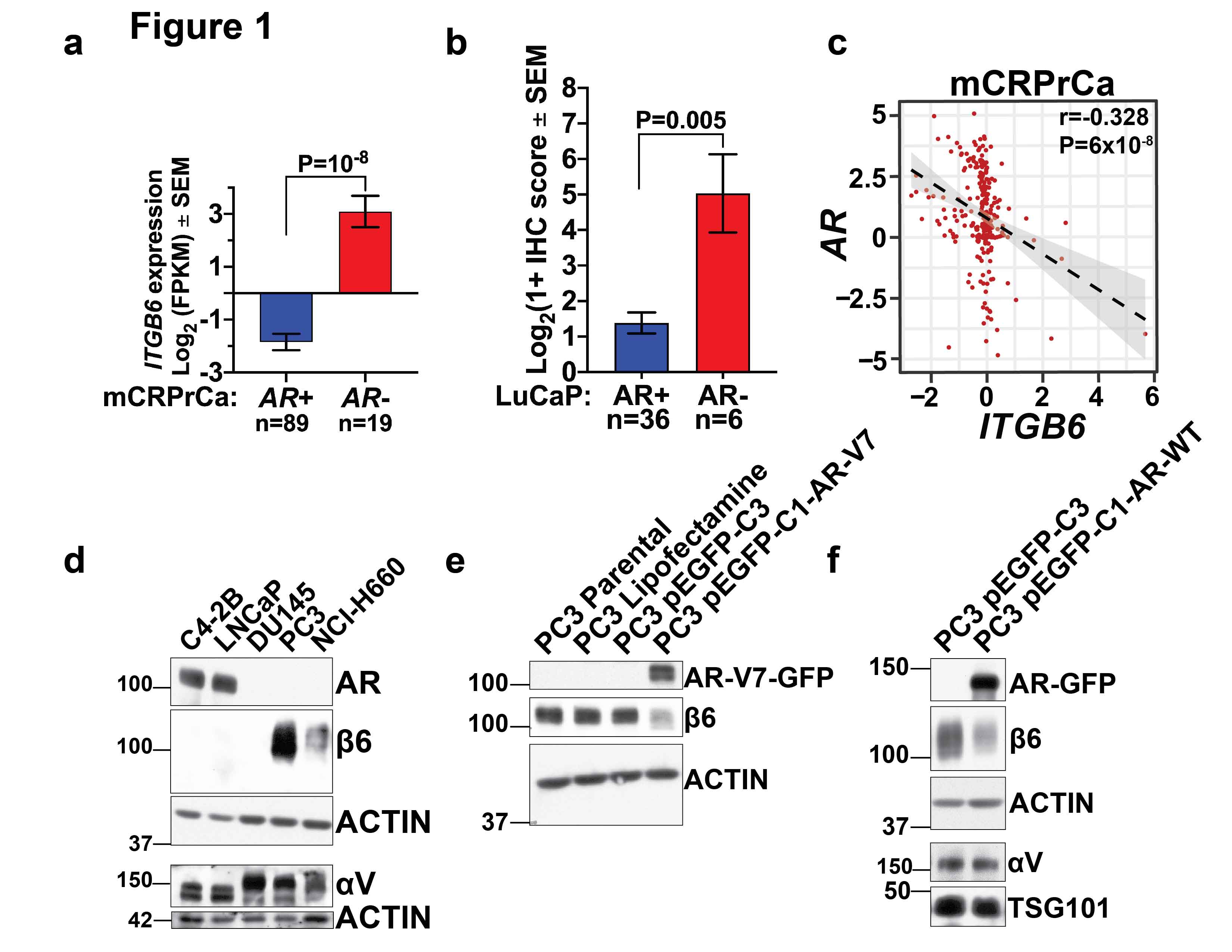

Supplement: Supplemental Material [file KCBT_A_2030622_SM7787.zip › supplementary/Final_12.23.21_Supplementary_Figure_1_2 (1).jpg]
